# The Moderation of Middle-Generation Support on the Relation of Stress and Depression in Coresident Grandparents

**DOI:** 10.1093/geroni/igab046.1005

**Published:** 2021-12-17

**Authors:** Danielle Nadorff, Melissa Barnett, Loriena Yancura

**Affiliations:** 1 Mississippi State University, Mississippi State, Mississippi, United States; 2 University of Arizona, Tucson, Arizona, United States; 3 University of Hawaii at Manoa, Honolulu, Hawaii, United States

## Abstract

Consistent with Cohen & Wills’ Buffering Hypothesis, social support has been found to moderate the relation between stress and depressive symptoms but has yet to be examined among coresident grandparents (CGPs), a population at risk of increased stress and depression. The current study sought to extend the model to this highly prevalent, vulnerable population. Participants were 180 grandparents across the USA living with their grandchildren. Measures included depression, stress, and satisfaction with support provided by the middle generation (MG) parent of the grandchild. After controlling for age, gender, income, and household type (skipped or multi-gen), MG support moderated the relation between perceived stress and depressive symptoms, accounting for 49% of variance. For CGPs least satisfied with support provided by the MG, the more stress, the higher their depressive symptoms. These findings indicate that improving relationships with grandchildren’s parents is an important avenue for interventions focused on grandparent caregivers’ mental health.

